# Validity and Reliability of Enzyme Immunoassays Using *Leishmania major* or *L. infantum* Antigens for the Diagnosis of Canine Visceral Leishmaniasis in Brazil

**DOI:** 10.1371/journal.pone.0069988

**Published:** 2013-07-26

**Authors:** Mauro Maciel de Arruda, Fabiano Borges Figueiredo, Fernanda Alvarenga Cardoso, Roberto Mitsuyoshi Hiamamoto, Júlia Cristina Macksoud Brazuna, Maria Regina Fernandes de Oliveira, Elza Ferreira Noronha, Gustavo Adolfo Sierra Romero

**Affiliations:** 1 Núcleo de Medicina Tropical, Universidade de Brasília, Brasília, Distrito Federal, Brazil; 2 Laboratório de Pesquisa Clínica em Dermatozoonoses em Animais Domésticos, Instituto de Pesquisa Clínica Evandro Chagas, Fundação Oswaldo Cruz, Rio de Janeiro, Rio de Janeiro, Brazil; 3 Fundação Ezequiel Dias-FUNED, Belo Horizonte, Minas Gerais, Brazil; 4 Instituto Adolfo Lutz, São Paulo, São Paulo, Brazil; 5 Centro de Controle de Zoonoses do Município de Campo Grande, Campo Grande, Mato Grosso do Sul, Brazil; 6 National Institute for Science and Technology for Health Technology Assessment (IATS/CNPq), Porto Alegre, Rio Grande do Sul, Brazil; National Central University, Taiwan

## Abstract

**Background:**

American visceral leishmaniasis is caused by the protozoan *Leishmania infantum.* Dogs are the main reservoirs in the domestic transmission cycle. The limited accuracy of diagnostic tests for canine leishmaniasis may contribute to the lack of impact of control measures recommended by the Brazilian Ministry of Health. The objective of this study was to estimate the accuracy of two enzyme-linked immunosorbent assays employing *L. major* or *L. infantum* antigens and their reliability between three laboratories of different levels of complexity.

**Methods:**

A validation study of ELISA techniques using *L. major* or *L. infantum* antigens was conducted. Direct visualization of the parasite in hematoxylin/eosin-stained histopathological sections, immunohistochemistry, and isolation of the parasite in culture.were used as gold standard. An animal that was positive in at least one of the tests was defined as infected with *L. infantum*. Serum samples collected from 1,425 dogs were analyzed. Samples were separated in three aliquots and tested in three different laboratories. Sensitivity, specificity and the area under de ROC curve were calculated and the reliability was evaluated between the participant laboratories.

**Results:**

The sensitivity was 91.8% and 89.8% for the *L. major* and *L. infantum* assays, respectively. The specificity was 83.75% and 82.7% for the *L. major* and *L. infantum* assays, respectively. The area under de ROC curve was 0.920 and 0.898 for *L. major* and *L. infantum*, respectively. The mean intraclass correlation coefficients between laboratories ranged from 0.890 to 0.948 when *L. major* was used as antigen, and from 0.818 to 0.879 when *L. infantum* was used.

**Interpretation:**

ELISA tests using *L. major* or *L. infantum* antigens have similar accuracy and reliability. Our results do not support the substitution of the *L. major* antigen of the ELISA test currently used for the diagnosis of canine visceral leishmaniasis in Brazil.

## Introduction

Visceral leishmaniasis (VL) is a severe parasitic disease that affects the phagocytic mononuclear system of humans and animals. In Brazil, the infection is caused by *Leishmania (Leishmania) infantum* (syn. *Leishmania (Leishmania) chagasi* [1], [2]. The parasite is transmitted mainly by the bite of female sandflies (*Lutzomyia longipalpis*) [2], [3], [4]. VL is endemic in Brazil and urbanization of the disease has been observed since 1980 [5]. Foci of VL are found in different cities of the five political-administrative regions of the country [6]. The prevalence of infected dogs that live in endemic areas ranges from 1% to 67% [3], [6].

One of the control measures of human VL recommended by the Brazilian Ministry of Health is euthanasia of seroreactive dogs in order to interrupt the transmission cycle [7]. Although employed since the 1950s, this measure continues to be controversial mainly because of its low effectiveness in reducing the incidence of canine and human disease [8], [9]. One of the factors that might be related to the lack of impact of this control measure is the limited accuracy of the tests used for the diagnosis of VL in dogs [10]. The Brazilian program uses two serological techniques for the diagnosis of canine VL: the enzyme-linked immunosorbent assay (ELISA) and the indirect immunofluorescent antibody test (IFAT). The diagnostic kits are supplied to the Central Public Health Laboratories (LACEN) of the country and to accredited laboratories that perform the tests in areas of VL transmission. The Ministry of Health recommends serological screening of the dogs with the ELISA and confirmatory diagnosis based on the IFAT results [7].

One of the disadvantages of serological tests is the possibility of false-positive results due to cross-reactions with other members of the family Trypanosomatidae, such as *Trypanosoma cruzi*, because of the existence of common epitopes that interfere with the specificity of the assays [11], . Specific tests are important to rule out VL in suspected clinical cases, whereas sensitive tests are fundamental for surveillance programs or to test dogs imported from endemic regions and to identify infected healthy animals [14].

According to Kar (1995) and Boarino (2008), the sensitivity and specificity of serological diagnostic methods depend on the type and purity of the antigen used [15], [16]. The tests currently available within the public health laboratory network in Brazil are produced by Bio-Manguinhos®, Fundação Oswaldo Cruz, Ministry of Health, and use *L. major* as antigen. However, there is growing technical and academic discussion about the possibility to improve the accuracy of the test by using *L. infantum* homologous antigen. This approach could increase the efficiency of culling seroreactive dogs and, consequently, the impact of the control program. We conducted the present study to answer wether a homologous crude antigen prepared with *L. infantum* would be able to improve the specificity of a crude antigen ELISA test currently prepared with *L. major*. Therefore, the objective of the present study was the validation and evaluation of reliability between laboratories with different complexity levels of two ELISA tests using *L. major* or *L. infantum* antigens.

## Methods

A validation study of ELISA techniques using *L. major* and *L. infantum* antigens for the diagnosis of canine VL was conducted. Parasitological tests were used as gold standards. In addition, the reliability between laboratories was tested by estimating intraclass correlation coefficients (ICC).

Serum and intact skin or skin lesion samples were collected from 1,600 dogs between 2008 and 2010 in a multicenter study conducted in four cities endemic for canine VL: Bauru, State of São Paulo; Brasília, Federal District; Palmas, State of Tocantins, and Fortaleza, State of Ceará, located in the southeast, center-west, north, and northeast regions of Brazil, respectively. Three neighborhoods with a historical prevalence of canine VL of 10% or higher were chosen in each city. The animals were selected without prior clinical assessment or laboratory diagnosis. The dogs were included based on systematic random sampling of the dwellings per street in each selected neighborhood. Dwellings were selected alternately from the first residence until a sample of 400 dogs was obtained from each of the four cities.

The skin samples were used for detection of the parasite by direct visualization in hematoxylin/eosin-stained histopathological sections, for immunohistochemistry according to Figueiredo et al. [17], and for isolation of the parasite in culture according to Madeira et al. [18]. The samples were processed at the Laboratory of Leishmaniasis Surveillance, Evandro Chagas Research Institute, Oswaldo Cruz Foundation (IPEC/FIOCRUZ, Rio de Janeiro), a National Referral Center for the parasitological diagnosis of leishmaniases. These techniques represented the gold standard and an animal that was positive in at least one of the tests was defined as infected with *L. infantum*.

Two ELISA tests produced by Bio-Manguinhos® were validated: ELISA using crude *L. major* antigen, a registered product of the Ministry of Agriculture and Livestock (MAPA) currently used by official diagnostic laboratories, and ELISA using crude *L. infantum* antigen, a pilot product produced by the same manufacturer which is not commercially available.

The tests were validated simultaneously in March and April 2010 by the National referral laboratory at the Ezequiel Dias Foundation in the State of Minas Gerais (FUNED-MG) and the reliability was evaluated by three referral laboratories of different levels of complexity that perform routine diagnostic tests of canine VL: the National Referral Laboratory at the Ezequiel Dias Foundation in the state of Minas Gerais (FUNED-MG), the State Referral Laboratory at the Adolfo Lutz Institute in the state of São Paulo (IAL-SP), and the Municipal Referral Laboratory at the Zoonosis Control Center in the municipality of Campo Grande, state of Mato Grosso do Sul (CCZ-CG). The tests were performed blindly by each laboratory, with the examiners being unaware of the result of the gold standard. The sera were cryopreserved at IPEC/FIOCRUZ and sent on dry ice to FUNED-MG with coded identification. The samples were then thawed and divided into 100-µL aliquots in eppendorf tubes. The tubes were sent under refrigeration in reusable ice to IAL-SP and CCZ-CG for the reliability assays.

The ELISA protocols used by the three laboratories were identical and were based on recommendations of the manufacturer of the diagnostic kit (Bio-Manguinhos®, Rio de Janeiro, Brazil). The results of the assays were read with routine equipment of the participating laboratories: a microplate spectrophotometer equipped with a 450-nm filter without the use of a reference filter (620–630 nm). The cut-off value was twice the mean optical density of the negative controls included in the plate according to manufacturer recommendations. Samples that presented an optical density between the cut-off and 1.2 times the cut-off were classified as indeterminate and re-tested. Samples that continued to be indeterminate were classified as negative. For standardization of the optical densities of the samples, the optical density obtained for the sample was divided by the respective cut-off. The product of this division was called the optical density index (ODI) and was used for evaluation of the reliability between laboratories based on the calculation of ICC.

The results were entered into Excel spreadsheets and analyzed using the SPSS 16 for Windows program. The following parameters were estimated: sensitivity, specificity, area under the ROC curve, positive (PPV) and negative predictive values (NPV), and ICC as a measure of reliability. A two-way random model for absolute agreement was used for calculation of the ICC. The respective 95% confidence intervals (95% CI) were estimated. Finally, a sensitivity analysis was performed to estimate the predictive values based on plausible seroprevalence rates previously reported in the literature [19], [20], [21], [22], [23], [24], [25].

### Ethics Statement

The project was approved by the Ethics Committee on Animal Use of Oswaldo Cruz Foundation (FIOCRUZ-CEUA) according to the Ethical Principles in Animal Research adopted by the Brazilian College of Animal Experimentation (COBEA), licensed under the number: G-38/08. All animal owners who participated in the experiment agreed to include their dogs in the study and signed an informed consent.

## Results

Of the 1,600 serum samples, 150 were excluded from the analysis because of inconclusive parasitological results obtained with the gold standards at IPEC/FIOCRUZ and 25 because the material was insufficient to perform all serological tests at the three laboratories. Thus, the final sample consisted of sera from 1,425 dogs. Of these, 98 (6.9%) were classified as positive and 1,327 (93.1%) as negative by the gold standard.

**Figure 1:**
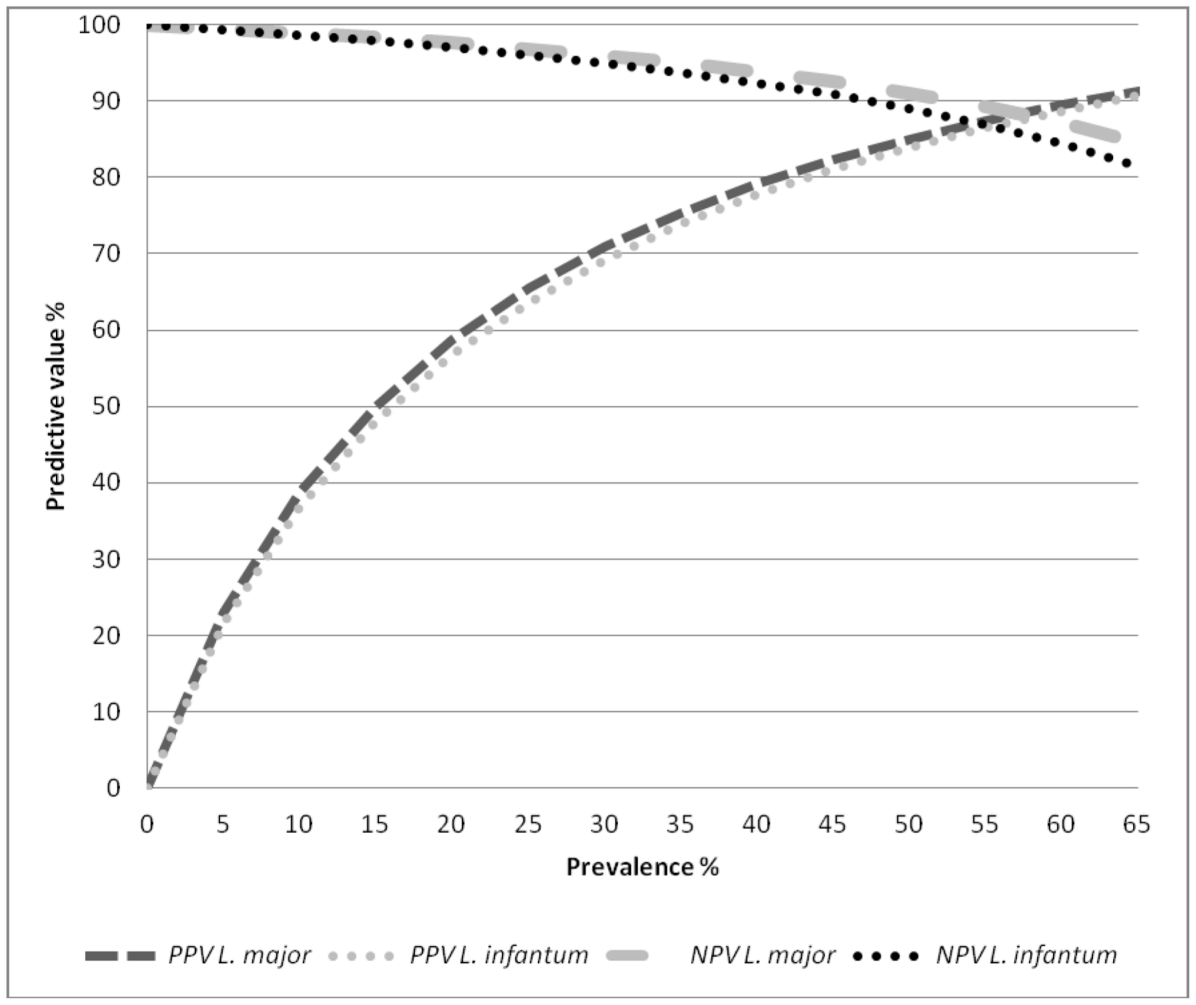
Sensitivity analysis of positive and negative predictive values of ELISA tests using *Leishmania major* or *Leishmania infantum* antigen (Bio-Manguinhos®) according to variations in the prevalence of canine visceral leishmaniasis. PPV: positive predictive value; NPV: negative predictive value.

The accuracy of the ELISA tests using *L. major* and *L. infantum* antigens in are shown in Table 1. Briefly, sensitivity was 91.84% and 89.80% and specificity was 83.57% and 82.59% for *L. major* and *L. infantum* antigens, respectively. Figure 1 shows the results of the sensitivity analysis for the calculation of NPV and PPV based on seroprevalence rates ranged from of 1 to 65%.

**Table 1:** Accuracy results of ELISA tests using *Leishmania major* or *Leishmania infantum* antigens (Bio-Manguinhos®) for the detection of visceral leishmaniasis in serum samples of dogs from endemic regions in Brazil (2011).

	*Leishmania major* test	*Leishmania infantum* test
Sensitivity	91.84% (86.42 to 97.26)	89.80% (83.80 to 95.79)
Specificity	83.75% (81.76 to 85.74)	82.69% (80.64 to 84.73)
AU_ROC_ a	0.917 (0.881 to 0.953)	0.893 (0.854 to 0.933)
PPV^b^	29.61% (24.47 to 34.74)	27.85% (22.91 to 32.79)
NPV^c^	99.28% (98.78 to 99.78)	99.09% (98.53 to 99.65)

The 95% confidence interval is given in parentheses.

a U_ROC_: area under the ROC curve.

b Positive predictive value.

c Negative predictive value.

**Figure 2:**
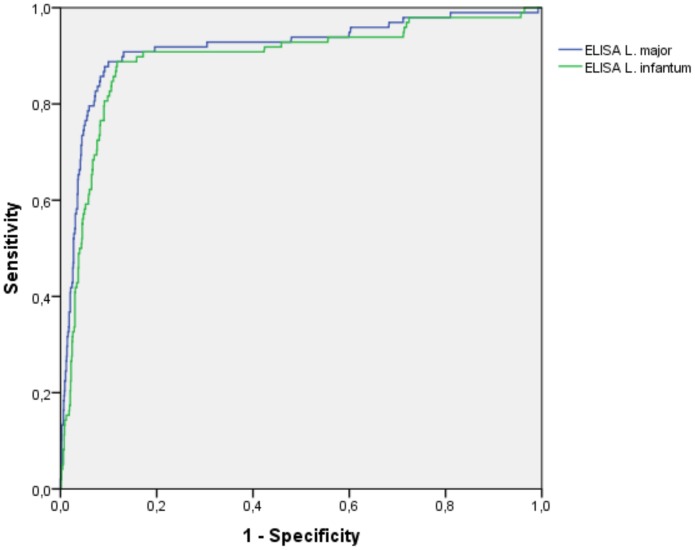
Receiver operating curve (ROC) comparing the results of the optic density indexes obtained with ELISA tests using *Leishmania major* or *Leishmania infantum* antigen (Bio-Manguinhos®).

The area under the ROC curve (AU_ROC_) was 0.920 and 0.898 for *L. major* and *L. infantum* antigens, respectively. Figure 2.

The mean ICC of the tests ranged from 0.890 to 0.948 when *L. major* was used as antigen, and from 0.818 to 0.879 when *L. infantum* was used. Table 2.

**Table 2:** Mean intraclass correlation coefficients between laboratories for the optic density indexes of ELISA tests using *Leishmania major* or *Leishmania infantum* antigen (Bio-Manguinhos®) for the detection of visceral leishmaniasis in serum samples of dogs from endemic regions in Brazil (2011).

Laboratory	*Leishmania major* test	*Leishmania infantum* test
	ICC^a^	ICC
CCZ-CG^b^ x IAL-SP^c^	0.904 (0.884 to 0.919)	0.818 (0.791 to 0.841)
IAL-SP x FUNED-MG^d^	0.948 (0.942 to 0.953)	0.879 (0.865 to 0.891)
CCZ-CG x FUNED-MG	0.890 (0.876 to 0.902)	0.875 (0.834 to 0.903)

The 95% confidence interval is given in parentheses.

a Intraclass correlation coefficient.

b Centro de Controle de Zoonoses, Campo Grande.

c Instituto Adolfo Lutz, São Paulo.

d Fundação Ezequiel Dias, Minas Gerais.

## Discussion

The relationship between human cases of VL and the prevalence of canine zoonotic disease has resulted in multiple efforts to reduce the risk of transmission. An important cornerstone of the national control program of the Brazilian Ministry of Health is the monitoring of reservoirs based on the identification and euthanasia of infected dogs. The development of accurate diagnostic tests that meet the needs of both public health services and owners of dogs living in endemic areas in terms of the reliability of a valid diagnosis is a major challenge for researchers.

The study of canine seroprevalence in endemic areas can generate much doubt depending on the sensitivity and specificity of the tests used, which vary between different validation studies. These variations are related mainly to differences in the reference population and sampling strategies used for the validation process, as well as to technical characteristics of the test, competence of the laboratory, choice of the gold standard, and cut-off value used for interpretation [26] **.** Furthermore, biological factors can affect the accuracy of serological tests. Sensitivity varies according to the state of infection and immune status of the host. In addition, lower specificity is due to cross-reactions with other agents or when this parameter is estimated in dogs that are truly infected but are not detected adequately by the gold standard [26]. Diagnostic tests based on recombinant more specific antigens have been developed, however the lack of sensitivity precludes their use as the first choice tools for epidemiological surveys or control intervention programs.

In view of these considerations, the present study evaluated the accuracy and reproducibility of Bio-Manguinhos® ELISA tests using *L. major* and *L. infantum* antigens for the detection of VL in serum samples of a random sample of dogs including the whole spectrum of *Leishmania* infection, from asymptomatic to seriously ill animals which represents the reality of canine VL in Brazil. The random sampling of dogs contributed to reduce selection bias, which is commonly seen in validation studies due to an unbalanced representation of symptomatic dogs. Another strong point of this study is the technical rigor and completeness of gold standard methods. However, our gold standard could fail in asymptomatic infected dogs with lower parasite burden producing a classification bias which would underestimate the true specificity value for both tests.

Comparison of the sensitivity between both antigens showed similar performance. Barbosa-De-Deus et al. [27] reported 98% sensitivity and 95% specificity of an ELISA test using antigen prepared from *L. major*-like promastigotes in a sample of 1,741 animals (1,582 negative and 159 positive). Similar results (97% sensitivity and 98% specificity) have been reported by Scalone et al. [28] for a sample of 415 animals (258 negative and 157 positive ) using recombinant rK39 antigen. Carvalho et al. [29] observed 100% sensitivity and specificity of an *in-house* ELISA using *L. infantum* antigen in 125 animals (15 negative and 110 positive ). The fact that another serological test was used as the gold standard in those studies might be a disadvantage since the tests evaluated would detect the same phenomenon of antibody elevation identified by the gold standard and the chance of agreement would therefore be higher, overestimating sensitivity and specificity.

Studying ELISA tests that employed *L. infantum/chagasi* antigen, Oliveira et al. [30] reported 90% sensitivity and 100% specificity of the test for a sample of 101 dogs, including 30 animals with a confirmed parasitological diagnosis and 71 negative animals. The authors used exclusively sera from dogs with a confirmed parasitological diagnosis for the calculation of sensitivity and exclusively sera from dogs defined as negative for the calculation of specificity, an approach that improves artificially the accuracy of the tests. Rosário et al. [13] compared ELISA tests employing crude antigens of *L. amazonensis* and *L. chagasi/infantum* and the recombinant antigens rK39 and rK26. A total of 131 samples were tested (25 negative and 106 positive) demonstrating sensitivity of 100% (95% CI: 95.6 to 100) for *L. amazonensis*, 98% (92.7 to 99.7) for *L. chagasi/infantum*, 98.1% (92.7 to 99.7) for antigen rK39, and 99.1% (94.1 to 100) for antigen rK26. Specificity was 100% (83.4 to100) for *L. amazonensis*, 100% (83.4 to 99.7) for *L. chagasi/infantum*, 100% (83.4 to 100) for antigen rK39, and 96% (77.7 to 99.8%) for antigen rK26. Lira et al. [31] evaluated ELISA with *L. major*-like antigen (Bio-Manguinhos®), which is currently used by the Brazilian visceral leishmaniasis control program, in a sample of 41 animals (25 positive and 16 negative) and observed sensitivity of 72% (50.4 to 87.1) and specificity of 87.5% (60.4 to 97.8%). In addition to the imprecise estimates of that study, the authors used animals from unaffected areas as negative controls, a fact that may have favorably influenced the specificity results. Pinheiro et al. [32] compared ELISA tests using a recombinant cysteine proteinase (rLdccys1) and lysates of *L. chagasi* amastigotes and promastigotes as antigens. In that study, sensitivity was 98% (rLdccys1), 89% (amastigotes) and 86% (promastigotes), and specificity was 96%, 69% and 68%, respectively. Like Oliveira et al. [30], the authors used sera from dogs with a confirmed parasitological diagnosis (209 animals) for the calculation of sensitivity and sera from 68 animals classified as negative for the calculation of specificity, including 46 samples from dogs with other diseases. However, the dog sera were obtained by convenience sampling which is prone to selection bias.

With respect to the predictive values shown in Table 1, the NPV were high (99,28 and 99,09%) indicating excellent sensitivity of the tests. On the other hand, the PPV was 29,61% when the *L. major* antigen was used and 27.85% with the *L. chagasi* antigen. These results are a matter of concern since in cases in which the prevalence of infection is similar to that of the sample studied (6.9%), at least three dogs with a false-positive result would be eliminated per each truly infected animal. In this respect, although designed to improve the capacity for detection of infection, the gold standard used probably continues to be imperfect and does not identify some truly infected animals, with a consequent impact on specificity and PPV.

As can be seen in Figure 1, the predictive values of the ELISA tests are closely related to the prevalence of the disease. As already mention above prevalence of canine visceral leishmaniasis is variable and health decision-makers need to be aware of the expected predictive values using serological tests for control purposes. In this respect, sensitive tests are fundamental for surveillance and control programs of leishmaniasis since they permit the culling of a larger number of truly infected animals, whereas specific tests are more important for the confirmation of suspected clinical cases, being more relevant for veterinarians dedicated to individual animal care [14]. Barbosa-De-Deus et al. [27] studied an important convenience sample of dogs in which the “prevalence” of VL was 9.13% (159/1741), obtained good predictive values (100% NPV and 66% PPV). However, as discussed earlier the selection bias that may occur as a result of the use of another serological test as the gold standard should be taken into account when interpreting these results.

In our study the ICC indicated almost perfect agreement between tests (>0.81) in labs with different levels of complexity. This is a very relevant result because adequate reproducibility is essential to avoid unnecessary dog culling and optimize lab costs. The lack of significant differences in the accuracy and reliability of tests using *L. infantum* or *L. major* antigen indicates that there is no need to change the antigen composition of the enzyme immunoassay currently used in Brazil for the diagnosis of canine VL.
